# Association of lower plasma citric acid with prolonged cough: the Nagahama study

**DOI:** 10.1038/s41598-023-40878-z

**Published:** 2023-08-25

**Authors:** Satoru Terada, Hisako Matsumoto, Kenta Nishi, Mariko Kogo, Natsuko Nomura, Noriyuki Tashima, Chie Morimoto, Hironobu Sunadome, Tadao Nagasaki, Tsuyoshi Oguma, Yoshinari Nakatsuka, Kimihiko Murase, Takahisa Kawaguchi, Yasuharu Tabara, Kazuhiro Sonomura, Fumihiko Matsuda, Kazuo Chin, Toyohiro Hirai

**Affiliations:** 1 Department of Respiratory Medicine, Kyoto University Graduate School of Medicine, Kyoto, Japan; 2 Department of Respiratory Medicine and Allergology, Faculty of Medicine, Kindai University, 377-2 Ohno-Higashi, Osakasayama City, Osaka Japan; 3 Department of Respiratory Care and Sleep Control Medicine, Kyoto University Graduate School of Medicine, Kyoto, Japan; 4 Center for Genomic Medicine, Kyoto University Graduate School of Medicine, Kyoto, Japan; 5 Graduate School of Public Health, Shizuoka Graduate University of Public Health, Shizuoka, Japan; 6 Technology Research Laboratory, Life Science Research Center, Shimadzu Corporation, Kyoto, Japan; 7 Department of Sleep Medicine and Respiratory Care, Division of Sleep Medicine, Nihon University of Medicine, Tokyo, Japan

**Keywords:** Respiratory signs and symptoms, Metabolomics

## Abstract

Little is known about the association of prolonged cough, a common and troublesome symptom, with metabolic pathways. We aimed to clarify this association using data from the Nagahama cohort, a prospective study of participants from the general population. Self-report questionnaires on prolonged cough were collected at baseline and 5-year follow-up assessments. Blood tests at follow-up were used for gas chromatography-mass spectrometry-based metabolomics. The association between metabolites and prolonged cough was examined using the partial least squares discriminant analysis and multiple regression analysis. Among the 7432 participants, 632 had newly developed prolonged cough at follow-up, which was defined as “new-onset prolonged cough”. Low plasma citric acid was significantly associated with new-onset prolonged cough, even after the adjustment of confounding factors including the presence of asthma, upper airway cough syndrome (UACS), and gastroesophageal reflux disease (GERD). A similar association was observed for isocitric acid, 3-hydroxybutyric acid, and 3-hydroxyisobutyric acid. The analysis of these four metabolites revealed that citric acid had the strongest association with new-onset prolonged cough. This significant association remained even when the analysis was confined to participants with UACS or GERD at baseline or follow-up, and these associations were also observed in participants (n = 976) who had prolonged cough at follow-up regardless of baseline status. In conclusion, low blood citric acid may be associated with prolonged cough.

## Introduction

Cough is one of the most common complaints of patients who seek medical attention, and a meta-analysis revealed that 9.6% of the general population had chronic cough^1^. Prolonged and chronic cough often impairs one’s quality of life; thus, elucidating the pathology of coughing and its prolongation is expected. Many diseases, such as cough variant asthma^2^, gastroesophageal reflux disease (GERD)^3^, and rhinosinusitis^4^, are well-known to often underlie prolonged cough. However, some participants complain of prolonged cough without these underlying diseases.

Irrespective of the presence of underlying diseases, patients with prolonged and chronic cough may have cough hypersensitivity syndrome, which is defined as “a clinical syndrome characterized by troublesome coughing that is often triggered by low thermal, mechanical, or chemical exposure levels”^5^. The anti-tussive effect of antagonists of P2X3 receptor that exists on C-fibers in the vagus nerve, may support the recently proposed paradigm of cough hypersensitivity syndrome^6^. Other potential mechanisms that underlie cough hypersensitivity syndrome include ion channels on C-fibers or airway sensory nerves, such as transient receptor potential (TRP) V1, V4, and A1^7^ and prostaglandin E2^8^. Furthermore, the airway sensory nerves can be activated with airway inflammation^9–11^ and oxidative stress^12^. However, the mechanisms that underlie prolonged or chronic cough remain largely unclear.

Metabolomics is a well-established metabolite analysis, including low molecular weight compounds, such as organic acids and amino acids. Metabolome’s measurement reflects the current phenotype of a particular biological system and is implemented to understand the pathophysiological processes. Metabolomics has been widely applied in the research of respiratory diseases, such as bronchial asthma, chronic obstructive pulmonary disease, and idiopathic pulmonary fibrosis^13^. Pathways related to hypoxic response, lipid metabolism, and tricarboxylic acid (TCA) cycle were identified in asthma^14,15^, but metabolites that are relevant to prolonged or chronic cough remain unknown. This study aimed to identify the metabolomics pathways that are related to prolonged or chronic cough.

## Results

**Figure 1:**
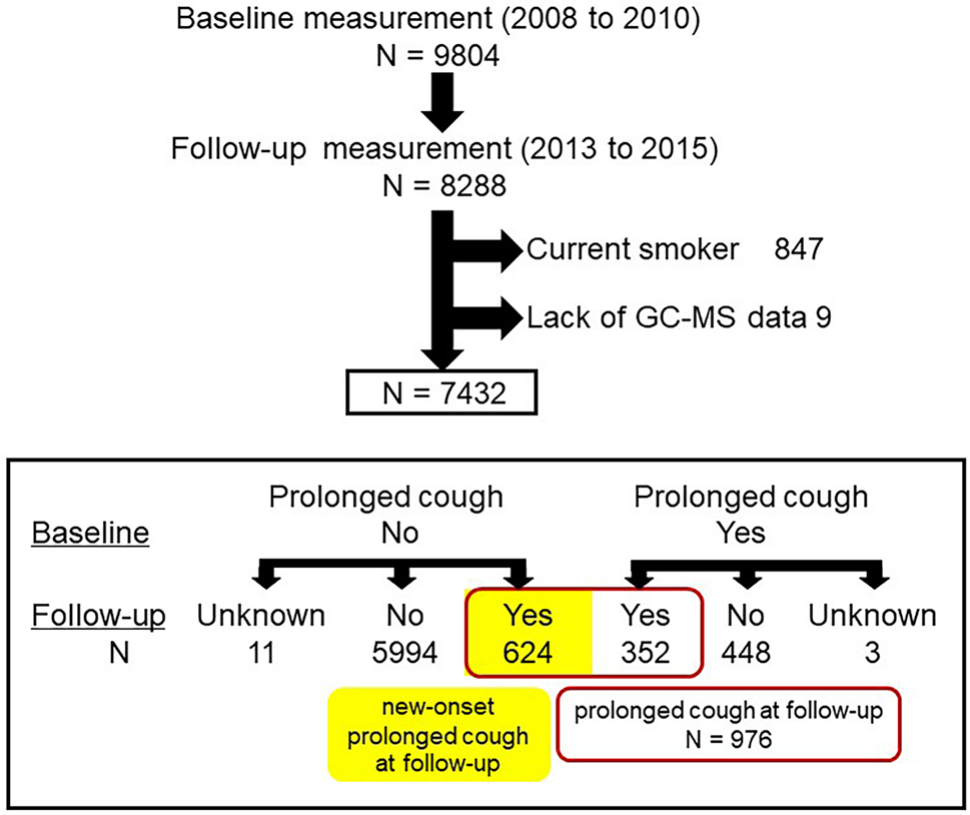
Flow of baseline and follow-up measurement of Nagahama cohort. In the period of baseline, serum IgE were measured. In the period of follow-up, questionnaires about the presence of various diseases and triggers of cough, and other blood collection were conducted. Patients with new-onset prolonged cough are highlighted in yellow, and those with a prolonged cough at follow-up are marked with a red line.

**Figure 2:**
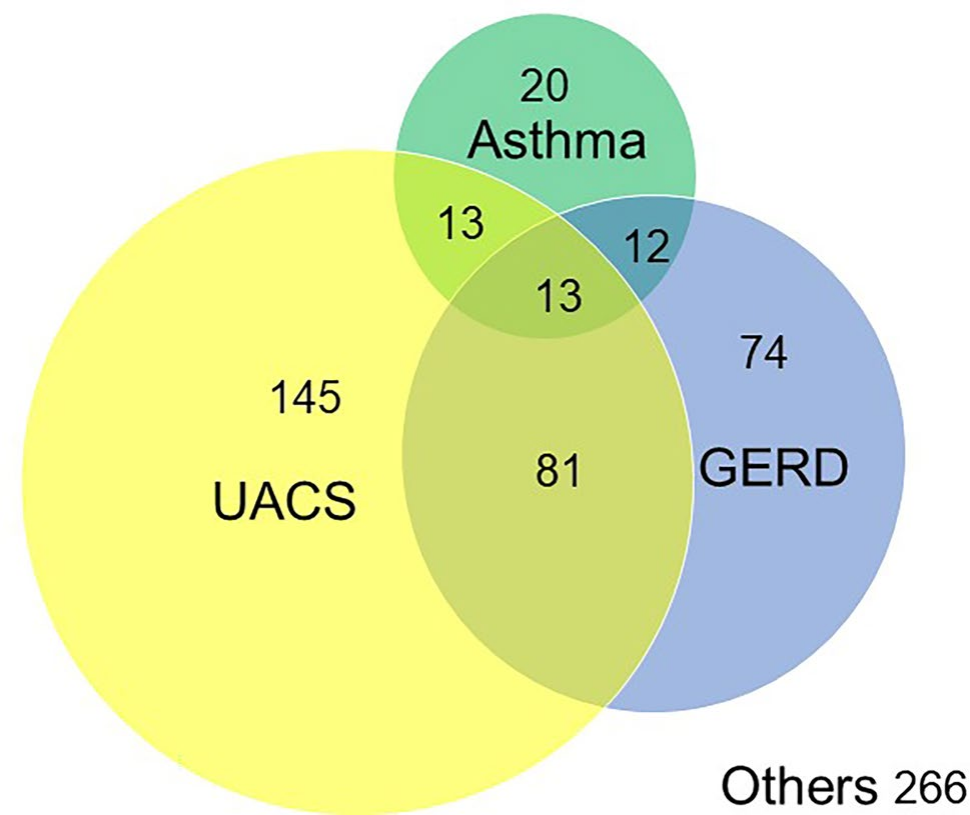
Underlying disease with new-onset prolonged cough group at follow-up.

Among the 7432 participants who were included in this study, 624 (9.4%) had a new-onset prolonged cough and 976 participants had a prolonged cough at follow-up (Fig. 1). Clinical characteristics of the participants who had a new-onset prolonged cough are shown in Table 1. The majority of the participants who had a new-onset prolonged cough were females and had higher frequency of asthma, upper airway cough syndrome (UACS), and GERD than those who did not have prolonged cough at any time (n = 5994). Overlapping of underlying diseases of prolonged cough, i.e., asthma, UACS, and GERD, in participants with a new-onset prolonged cough is presented in Fig. 2 and Table S2. A total of 119 participants had two or more diseases, whereas 266 participants did not have these underlying diseases. Additionally, 38% of participants with a new-onset prolonged cough were in a depressive state, which was significantly higher than the remaining (28%) (*p* < 0.001).

**Table 1:** Patient characteristics of new-onset prolonged cough at follow-up using comparative and multiple regression analyses.

	Comparative analysis			Multivariate analysis	
	New-onset prolonged cough		*P*-value	OR (95% CI)	*P*-value
	Yes	No			
	N = 624	N = 5994			
Age, years	61 ± 13	63 ± 13	0.003	0.98 (0.91–1.05)	0.58
Sex, female, %	79	71	< 0.001	1.38 (1.06–1.80)	0.02
BMI, kg/m^2^	21.7 ± 3.4	21.8 ± 3.2	0.77	1.01 (0.96–1.07)	0.74
Ex smoking, %	20	25	0.003	0.83 (0.64–1.08)	0.16
%FEV_1_, %	103 ± 15	103 ± 16	0.44	0. 95 (0.90–1.01)	0.08
Serum total IgE, IU/mL*	56 ± 436	64 ± 728	0.06	0.81 (0.68–0.95)	0.009
Blood eosinophil count, /μL*	109 ± 110	103 ± 140	0.92	1.00 (0.99–1.01)	0.92
Asthma, %	9	4	< 0.001	2.51 (1.78–3.55)	< 0.001
Upper airway cough syndrome, %	40	22	< 0.001	2.15 (1.79–2.58)	< 0.001
FSSG, ≧ 8, %	29	20	< 0.001	1.45 (1.19–1.76)	< 0.001
Citric acid, intensity*	0.66 ± 0.13	0.68 ± 0.14	0.0002†	‡	‡

Data are presented as mean ± standard deviation. Serum IgE level was measured at baseline, and *Log-transformed value was used for analysis. †*p* value after Bonferroni correction. ‡see Table 2.

*OR* odds ratio, *CI* confidence interval, *BMI* body mass index, *FEV*_*1*_ forced expiratory volume in one second, *FSSG* frequency scale for the symptom of GERD, *GERD* gastroesophageal reflux disease.

For multivariate analysis, age per 10-year increase, BMI per 2 kg/m^2^, ex smoking, Blood eosinophil count per 10/μL,　and %FEV_1_ per 10% were used.

**Figure 3:**
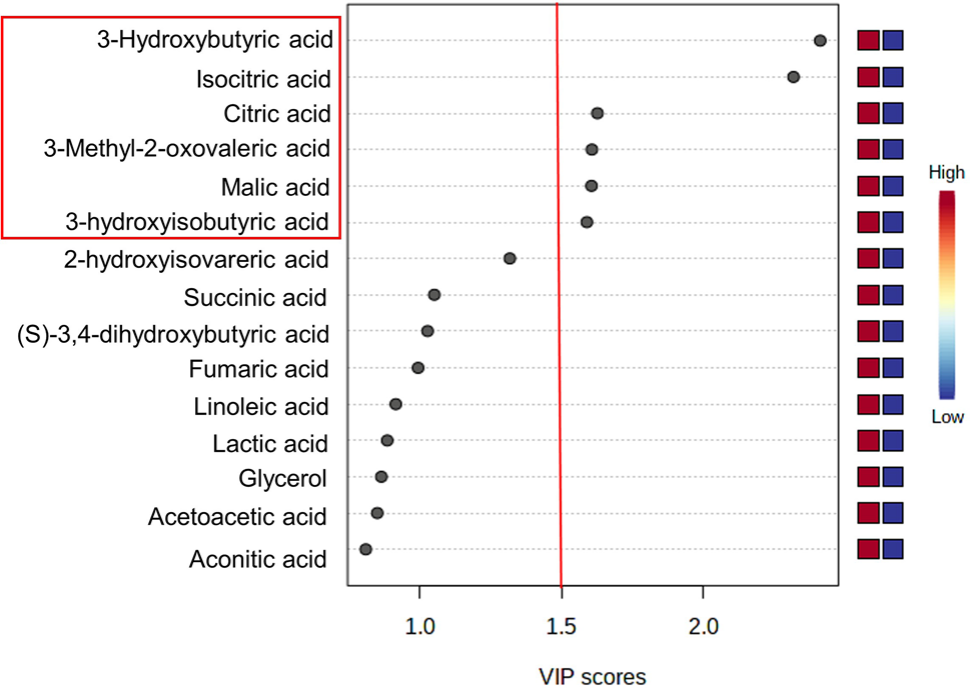
Variable importance projection (VIP) score calculated from Partial least-squares discriminant analysis (PLS-DA). VIP scores of metabolite profiles generated by GC–MS data of new onset prolonged cough at follow-up measurement by using Metaboanalyst 4.0 were measured. Heat map with red or blue boxes indicates high or low abundance ratio, respectively, of metabolites with new-onset prolonged cough. 3-hydroxybutyric acid, isocitric acid, citric acid, 3-methyl-2-oxovareric acid, malic acid, and 3-hydroxybutyric acid showed VIP score 1.5 or over. PLS-DA: Partial least squares discriminant analysis, VIP: Variable importance in projection.

Partial Least Squares Discriminant Analysis (PLS-DA) for a new-onset prolonged cough yielded 6 metabolites that had the variable importance in projection　scores of 1.5 or more; 3-hydorxybutyric acid (BHB), isocitric acid, citric acid, 3-methyl-2-oxovaleric acid, malic acid and 3-hydroxyisobutyric acid (HIBA) (Fig. 3). In a comparative analysis, citric acid level was significantly lower in participants with new-onset prolonged cough than in those without new-onset prolonged cough (*p* = 0.0002 after Bonferroni correction) (Table 1). Lower levels of plasma citric acid, isocitric acid, BHB, and HIBA, but not malic acid or 3-methyl-2-oxovaleric acid, were significantly associated with a new-onset prolonged cough in analyses adjusted with various factors, including %FEV_1_ and the presence of asthma, UACS, and GERD (Table 2). Similarly, these metabolites were also associated with a prolonged cough at follow-up in the adjusted models (data not shown).

**Table 2:** Adjusted odds ratio of each metabolite for new-onset prolonged cough at follow-ups.

Metabolites	New-onset prolonged cough at follow-up	
	OR (95% CI)	*P*-value
Citric acid, intensity	0.11 (0.04–0.31)	< 0.001
Isocitric acid, intensity	0.36 (0.18–0.74)	0.005
Malic acid, intensity	0.41 (0.16–1.07)	0.07
BHB, intensity	0.70 (0.56–0.88)	0.002
HIBA, intensity	0.45 (0.23–0.87)	0.02
3-methyl-2-oxovaleric acid, intensity	0.49 (0.22–1.08)	0.08

Adjusted with factors at follow-up except for serum total IgE: age, sex, BMI, ex smoking, %FEV_1_, serum total IgE at baseline, blood eosinophil count, upper airway cough syndrome, asthma, FSSG, and blood collection time after meals. Serum IgE level, blood eosinophil count, and 6 metabolites were log-transformed. ORs were calculated per one unit increase in intensity.

*OR* odds ratio, *CI* confidence interval, *BHB* 3-hydroxybutyric acid, *HIBA* 3-hydroxyisobutyric acid.

**Figure 4:**
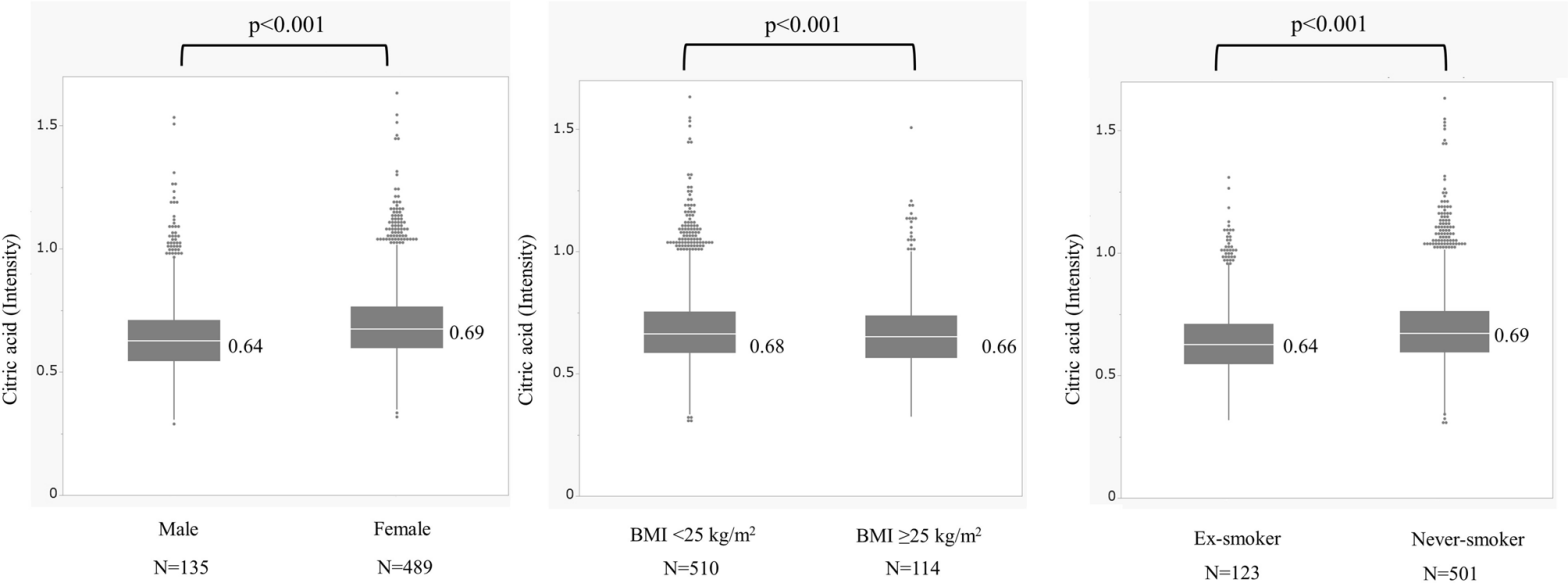
Univariate analysis of plasma citrate in association with (**a**) sex, (**b**) body mass index (BMI) at follow-up, and (**c**) smoking status. Citrate concentration was log transformed.

The associations between these metabolites were at most moderate; Pearson’s correlation coefficients between citric acid and isocitric acid, citric acid and BHB, and citric acid and HIBA were 0.57, 0.45, and 0.16, respectively. Therefore, the four metabolites were evaluated in a multivariate analysis altogether (Table 3). Only the lower citric acid level was significantly associated with a new-onset prolonged cough, and with a prolonged cough at follow-up, independent of asthma, UACS, and GERD. Although there was a significant difference in plasma citric acid among the six groups that are classified during the postprandial period (Figure S1), which was the highest at 5–9 h after meals, adjustment with postprandial period did not change the result. In participants with new-onset prolonged cough, males, overweight participants, ex-smokers (Fig. 4) and younger participants (data not shown) showed lower plasma citric acid than their counterparts.

**Table 3:** Multiple regression analysis of new-onset prolonged cough and prolonged cough at follow-up involving 4 metabolites.

	New-onset prolonged cough		Prolonged cough at follow-up	
	OR (95% CI)	*P*-value	OR (95% CI)	*P*-value
Age, per 10-yr increase	0.98 (0.90–1.05)	0.54	0.92 (0.86–0.98)	0.006
Sex, female	1.37 (1.04–1.81)	0.02	1.40 (1.12–1.75)	0.004
BMI, per 2 kg/m^2^	1.01 (0.95–1.07)	0.71	1.02 (0.97–1.07)	0.51
Ex smoking, %	0.84 (0.65–1.09)	0.18	0.81 (0.66–1.01)	0.06
%FEV_1_, per 10%	0.95 (0.90–1.01)	0.08	0.96 (0.91–1.00)	0.07
Serum total IgE at baseline, IU/mL*	0.81 (0.69–0.95)	0.01	0.82 (0.72–0.94)	0.004
Blood eosinophil count, per 10 IU/μL*	1.00 (0.99–1.01)	0.68	1.00 (0.99–1.00)	0.30
Asthma	2.53 (1.79–3.57)	< 0.001	2.99 (2.31–3.88)	< 0.001
Upper airway cough syndrome	2.14 (1.79–2.57)	< 0.001	2.03 (1.74–2.36)	< 0.001
FSSG, ≥ 8	1.45 (1.19–1.76)	< 0.001	1.52 (1.30–1.79)	< 0.001
Citrus consumption	1.01 (0.81–1.27)	0.91	0.92 (0.77–1.12)	0.41
Blood collection time after meal, per 1 h	0.98 (0.96–1.01)	0.17	0.99 (0.98–1.01)	0.55
Citric acid, intensity*	0.18 (0.04–0.78)	0.02	0.17 (0.05–0.58)	0.004
Isocitric acid, intensity*	0.83 (0.33–2.09)	0.70	1.26 (0.59–2.70)	0.56
BHB, intensity*	0.83 (0.61–1.12)	0.20	0.83 (0.64–1.07)	0.14
HIBA, intensity*	1.04 (0.43–2.53)	0.92	1.07 (0.51–2.21)	0.86

*Log-transformed value was used for analysis.

*OR* odds ratio, *CI* confidence interval, *BMI* body mass index, *FEV*_*1*_ forced expiratory volume in one second, *FSSG* frequency scare for the symptom of GERD, *GERD* gastroesophageal reflux disease, *BHB* 3-hydroxybutyric acid, *HIBA* 3-hydroxyisobutyric acid. ORs were calculated per one unit increase in intensity.

The risks of lower plasma citric acid for a new-onset prolonged cough and prolonged cough at follow-up remained significant when the analysis was confined to participants with UACS or GERD at baseline or follow-up (data not shown). When the analysis was confined to participants with asthma, the new-onset prolonged cough was associated with lower citric acid after adjustment with age, sex, body mass index, ex smoking, %FEV_1_, serum total Immunoglobulin (Ig)E, blood eosinophil count, and blood collection time after meals (data not shown). Additionally, lower plasma citric acid was also significantly associated with both new-onset prolonged cough and prolonged cough at follow-up when we excluded participants who had chronic kidney disease (n = 990) or osteoporosis (n = 643) (data not shown), which might have affected the plasma citric acid level^16,17^.

Lastly, the role of plasma citric acid for cough continuation in participants with cough triggers was examined. Among the 19 cough triggers, the top three were itchy throat, common cold, and dust exposure (Table S3). Lower plasma citric acid level was associated with new-onset prolonged cough and prolonged cough at follow-up among the 3375 subjects who had at least one of the 3 triggers without asthma, UACS, or GERD (Table S4a), even after adjustment with age, sex, body mass index, ex smoking, %FEV_1_, serum total IgE, blood eosinophil count, and blood collection time after meals (Table S4b).

## Discussion

To our best knowledge, this is the first report to show an association of plasma metabolites with prolonged cough in the general population. Additionally, low levels of plasma citric acid, isocitric acid, BHB, and HIBA were risk factors for prolonged cough in this large population-based study. Among the four metabolites, citric acid was the most closely associated with prolonged cough and independent of the presence of asthma, UACS, and GERD. Finally, low plasma citric acid level was a risk of new-onset prolonged cough in participants with cough-triggering factors in the absence of these 3 diseases.

This study revealed an association between lower citric acid and new-onset prolonged cough, independent of asthma, UACS, and GERD. Mechanisms underlying the associations between lower citric acid and prolonged cough remain unknown, but airway inflammation is well-known to increase in non-asthmatic patients with chronic cough. The inflammation includes neurogenic and contribution of various cells, such as eosinophils, lymphocytes, neutrophils, and mast cells^18–20^. Furthermore, Clark et al.^12^ recently revealed that mitochondrial reactive oxygen species activate airway sensory nerves, via TRP channels, and protein kinase C, and finally evoke cough reflexes. Citrate^21,22^ exhibits anti-inflammatory and antioxidative properties as discussed below; thus, suggesting that decreased citric acid may fail to protect from airway inflammation and oxidative stress is plausible, which in turn leads to prolonged cough (Figure S2a). Additionally, airway inflammation and oxidative stress are involved in the pathophysiology of major underlying diseases of chronic coughs, such as asthma^23^, chronic rhinosinusitis^24^, and GERD^25^; thus, decreased citric acid may accelerate cough in participants with underlying diseases.

Citrate exerted anti-inflammatory properties in lipopolysaccharide-stimulated RAW264.7 macrophages, which may or may not have sodium-coupled citrate transporter, by inhibiting interleukin (IL)-1β, IL-6, and tumor necrosis factor-α production^21^, as well as antioxidative properties by reducing the intracellular reactive oxygen species generation and increasing the antioxidant enzyme activities^21^. Similarly, treatment with citrate increased the levels of free radical scavenging enzymes and decreased pro-oxidant protein and pro-inflammatory factors in bone marrow mesenchymal stem cells, although citrate itself was not a free radical scavenger^26^. Furthermore, citrate exerts antioxidative effects as a metal chelate, particularly through its iron chelation activity, which may prevent the Fenton reaction that generates hydroxyl radicals in the presence of hydrogen peroxide and ferrous ion (Fe^2+^)^26^.

The mechanisms of decreased citric acid levels in participants with prolonged cough remain unknown. Citrus fruit intake was similar between participants with and without prolonged cough, and plasma citric acid is considered relatively independent of normal dietary citric acid intake^16,27^. Rather, blood citric acid level is affected by chronic kidney disease^16^ and metabolic bone diseases, such as osteoporosis^17^. However, this study revealed a significant contribution of low plasma citric acid to prolonged cough, even when participants with osteoporosis and chronic kidney diseases were excluded from the analysis. Another potential explanation is the down-regulation of TCA cycle activity in participants with prolonged cough. Generally, blood and urine levels of citric acid reflect TCA cycle activity^16^, which is reported to be modulated in several pulmonary diseases, such as asthma, chronic obstructive pulmonary disease, idiopathic pulmonary fibrosis, and particulate matter 2.5 exposure^14,15,28–30^. Indeed, other TCA cycle intermediates of isocitric acid and malic acid were also decreased in prolonged coughers in this study, albeit insignificantly for malic acid (Table 2). Reduced activity of TCA cycle may result in reduced ATP production in neurons, which might change the sensitivity of P2X3 receptor, an important receptor responsible for cough hypersensitivity, to ATP. Otherwise, a decrease in plasma citric acid can be explained by intracellular consumption to regulate mitochondrial oxidative stress as an inflammation consequence. Although the mechanisms remain unknown, lower blood citric acid could be a therapeutic target for prolonged cough. While citric acid inhalation is a foe, as it induces cough by its weak acidic nature, whether citric acid intake could be a friend to prolonged and chronic cough should be further examined.

The potential role of BHB that was decreased in prolonged coughers, albeit not as greatly as citric acid should also be discussed (Table 2, Figure S2b). BHB has an anti-inflammatory effect by reducing the NLR family pyrin domain containing 3 (NLRP3) inflammasome-mediated IL-1β and IL-18 production in human monocytes. In vivo, BHB attenuates caspase-1 activation and IL-1β secretion in mouse models of NLRP3-mediated diseases^31^. Yamanashi et al.^32^ revealed that BHB inhibits NLRP3-induced neuroinflammation in the hippocampus in rats of acute and chronic stress model. Thus, decreased BHB may not prevent neuroinflammation and may contribute to cough hypersensitivity.

Apart from the contribution of metabolites to prolonged cough, we found that the depressive status frequency was greater in coughers than in non-coughers (38 vs. 28%, *p* < 0.001). The previous report from Korea showed that chronic cough was associated with depression in elderly people without asthma^33^, and several surveys indicate that chronic persistent cough is accompanied by increased anxiety and depression symptoms^34,35^. Therefore, we examined the depressive status of participants with prolonged and chronic cough, and our results were consistent with previous reports^33–35^.

This study has several limitations. Although the prevalence of participants who had a new-onset prolonged cough at follow-up in this study was similar to the prevalence of chronic cough in other ethnicities (9.4%)^1^, we could not evaluate chronic cough alone, which is defined to last for 8 weeks or longer. In addition, cough-specific interviews were not conducted as part of the Nagahama study, which involved a large number of comprehensive questionnaires covering various organ diseases. Consequently, information regarding the presence of cough at the time of the interview, its recurrence or persistence, cough bout frequency, and impact on the participants' quality of life remains unknown. Approximately half of the participants with a new-onset prolonged cough or prolonged cough at follow-up reported having sputum symptoms. An analysis of the data revealed no significant relationship between plasma citric acid levels and the sputum symptoms (data not shown). In addition, this study included only residents who were willing to participate, and it is possible that potentially health-conscious participants may have been selected. Next, this analysis focused on plasma metabolites, and whether metabolite level in plasma reflects the intracellular dynamics of participants is unclear and further research is needed. Lastly, metabolite levels were measured only at follow-up; thus, determining a causal relationship with new-onset prolonged cough is impossible. It is also difficult to determine precisely when plasma citric acid dropped in patients with a history of prolonged cough because blood samples were taken after the onset of cough. Studies on changes in plasma citric acid following therapeutic interventions are warranted.

In conclusion, this study revealed that low plasma TCA cycle intermediates, particularly citric acid, were risk factors for prolonged cough in a large sample of the general population. Elucidating the roles of citric acid in the pathogenesis of prolonged/chronic cough, including the effects on the airway sensory nerve, is challenging but required future study.

## Methods

### Study design, population and measurements

The Nagahama prospective genome cohort is a population-based study of prolonged cough and various diseases. The subjects were participants of the Nagahama Cohort for Comprehensive Human Bioscience (the Nagahama study). The participants were recruited from the general population, aged 30–74 years, living in Nagahama city (approximately 125,000 inhabitants), in Shiga Prefecture in Japan. The baseline assessment that was conducted from 2008 to 2010 was voluntarily participated by 9804 residents. A follow-up survey was conducted 5 years after the baseline assessment from November 2013 to November 2015 on participants who had not waived their consent. Aside from serum total IgE, blood tests and pulmonary function tests at follow-up assessment were analyzed. Blood tests at follow-up were used for gas chromatography-mass spectrometry (GC–MS)-based metabolomics. Details of blood sampling for GC–MS are presented in the Supplementary file and Table S1 in the Supplemental material. The present study excluded current smokers and participants with missing essential GC–MS data. Finally, this study included 7432 participants.

### Structured questionnaires

The presence of prolonged cough was asked with this questionnaire at baseline and follow-up assessments:

Have you ever experienced a cough for 3 weeks or more?

As a result, the group identified as having a “prolonged cough” in this study encompassed individuals with a cough lasting for 3 to 8 weeks, as well as those with chronic cough persisting for 8 weeks or longer^36–38^. Participants who had newly developed prolonged cough at follow-up were defined as “new-onset prolonged cough” and those who had prolonged cough at follow-up regardless of baseline status as “prolonged cough at follow-up.”

The determination of asthma and postnasal drip presence was based on self-reported questionnaires, and the presence of postnasal drip was considered indicative of UACS. GERD was evaluated using the Frequency Scale for the Symptoms of GERD (FSSG)^39^, and participants who had an FSSG score of 8 or more were defined as having GERD. Additionally, we defined “depressive status” as scores of 16 or more following the Center for Epidemiologic Studies Depression Scale (CES-D)^40^, which is a screening test for depression. Citrus consumption and cough-triggering factors at follow-up were also examined.

### Statistical analysis

For the metabolome analysis, we performed PLS-DA using Metaboanalyst 4.0 (details are presented in the Supplemental file). For the general statistical analysis, data were analyzed using JMP version Pro 15 (SAS Institute Inc., Tokyo, Japan). Two groups were compared using a t-test or one-way analysis of variance, as appropriate. Logistic regression analyses were performed to investigate contributing factors to the new-onset prolonged cough and prolonged cough at follow-up. The Pearson’s correlation coefficient was used to test the strength of associations between the metabolites. The Bonferroni correction was performed to reduce the risk of type I errors associated with multiple comparisons of metabolites.

### Patient and public involvement statement

Citizens were not directly involved in the design of this study, but their opinions for the length and volume of the questionnaires were taken into account.

The Nagahama Zeroji Cohort is a collaboration between Nagahama City (government agency), Kyoto University, and a non-profit organization whose board members and members are mostly Nagahama citizens. The study results are available at https://zeroji-cohort.com/articles/. We thanked our patient advisors in our acknowledgements.

### Ethical approval

Study procedures were approved by the ethics committee of Kyoto University Graduate School and Faculty of Medicine and the Nagahama Municipal review Board. This research was performed in accordance with relevant guidelines/regulations. Written informed consent was obtained from all participants.

### Supplementary Information

Supplementary Information.

## Data Availability

The datasets generated and analyzed during the current study are not publicly available due to ethical restrictions but are available from the corresponding author on reasonable request.
