# Supplementary material for: Association of lower plasma citric acid with prolonged cough: the Nagahama study
Source: Sci Rep. 2023 Aug 25;13:13921. doi: 10.1038/s41598-023-40878-z (PMC10457296; doi:10.1038/s41598-023-40878-z)
Supplement: Supplementary file 1 — Supplementary Information. [file 41598_2023_40878_MOESM1_ESM.pdf]

Association of lower plasma citric acid with prolonged cough: The Nagahama Study

Short title: citric acid and prolonged cough

Satoru Terada, MD; Hisako Matsumoto, MD, PhD; Kenta Nishi, MD; Mariko Kogo, MD; Natsuko Nomura, MD; Noriyuki Tashima, MD; Chie Morimoto, MD, PhD; Hironobu Sunadome, MD, PhD; Tadao Nagasaki, MD, PhD; Tsuyoshi Oguma, MD, PhD; Yoshinari Nakatsuka, MD, PhD; Kimihiko Murase, MD, PhD; Takahisa Kawaguchi, PhD; Yasuharu Tabara, PhD, Kazuhiro Sonomura, PhD, Fumihiko Matsuda, MD, PhD, Kazuo Chin, MD, PhD; Toyohiro Hirai, MD, PhD

Supplemental Methods

Blood sampling for a metabolomics

The internal standard solution (2-isopropylmalic acid at 0.1 mg/mL in purified water) and extraction solvent (methanol: water: chloroform = 2.5:1:1) were mixed at a ratio of 6:250, which was added to 50 mL of each plasma sample. The resulting solution was mixed using a shaker at 1,200 rpm for 30 min at 37°C. After centrifugation at 16,000 × g for 5 min at 4°C, 150 mL of supernatant was collected and mixed with 140 mL of purified water. The solution was thoroughly mixed and centrifuged at 16,000 × g for 5 min at 4°C. Finally, 180 mL of supernatant was collected and lyophilized. The lyophilized sample was dissolved in 80 mL of methoxyamine solution (20 mg/mL in pyridine) and agitated at 1,200 rpm for 30 min at 37°C. Forty micro litters of N-methyl-N-trimethylsilyltrifluoroacetamide solution (GL Science, Tokyo, Japan) were added for trimethylsilyl derivatization, followed by agitation at 1,200 rpm for 30 min at 37°C. After centrifugation at 16,000 × g for 5 min at room temperature, 50 mL of supernatant was transferred to a glass vial and subjected to GC-MS measurement, which was conducted using GCMS-QP2010 Ultra (Shimadzu, Kyoto, Japan). The intensity of each metabolite was calculated as the ratio of peak area of the target substance to that of standard substance. Log-transformed value was used for analysis, since the intensity of each metabolite was not normally distributed.

Metabolome analysis

We performed Partial Least Squares Discriminant Analysis (PLS-DA) using Metaboanalyst 4.0 before the general statistical analysis. In this study, we selected 35 metabolites that have been previously

reported to be relevant in the metabolome analysis of chronic respiratory diseases[1-4], including carboxylic acids, such as fatty acids and their oxide and hydroxide, ketone bodies, tricarboxylic acid cycle intermediates, and it's up/downstream metabolites (Table 1). After log-transformation, 35 compounds, were mean-centered and divided by the standard deviation of each variable. The variable importance in projection (VIP) for new-onset prolonged cough was generated, which was a weighted sum of squares of the PLS loadings, in the PLS-DA considering the amount of explained Y-variation in each dimension. Our study defined metabolites with a VIP score of ≥1.5 as a candidate for independent risk metabolites.

References

1. Nambiar S *et al*. Metabolomics in chronic lung diseases. *Respirology* **25**, 139-148, doi: 10. 1111/resp. 13530 (2020)
2. Chang C *et al*. Metabolic alterations in the sera of Chinese patients with mild persistent asthma: a GC-MS-based metabolomics analysis. *Acta Pharmacol Sin* **36**, 1356-1366, doi: 10.1038/aps.2015.102 (2015)
3. Kelly RS *et al*. Asthma Metabolomics and the Potential for Integrative Omics in Research and the Clinic. *Chest* **151**, 262-277, doi: 10.1016/j.chest.2016.10.008 (2017)
4. Tashima, N *et al.* Evaluation of elevated plasma fatty acids as relevant factors for adult-onset asthma: The Nagahama Study. *Allergol Int,* doi: 10.1016/j.alit.2023.04.005 [published Online First: 2023/05/18).

Table S1. 35 metabolites were analyzed of PLS-DA

fatty acid and these oxide and hydroxide	2-hydroxybutyric acid
	3-hydroxybutyric acid
	2-hydroxyisobutyric acid
	3-hydroxyisobutyric acid
	2-hydroxyisovaleric acid
	3-hydroxyisovaleric acid
	(S)-3,4-dihydroxybutyric acid
	3-methyl-2-oxobutyric acid
	acetoacetic acid
	glycerol
	glycolic acid
	2-oxoisocaproic acid
	caproic acid
	caprylic acid
	decanoic acid
	lauric acid
	myristic acid
	palmitoleic acid
	margaric acid
	elaidic acid
	linoleic acid
	oleic acid
TCA intermediates	citric acid
	isocitric acid
	2-oxoglutaric acid (α-ketoglutaric acid)
	succinic acid
	fumaric acid
	aconitic acid
	lactic acid
	malic acid
	2-oxobutyric acid
	pyruvic acid
others	oxalic acid
	3-methyl-2-oxovaleric acid
	3-(3-hydroxyphenyl)-3-hydroxypropionic acid

PLS-DA: partial least squares-discriminant analysis, TCA: tricarboxylic acid

Table S2. Underlying diseases in participants with a new-onset prolonged cough at follow-up.

n	UACS (n=252)	GERD (n=180)	Asthma (n=58)
13	+	+	+
81	+	+	-
13	+	-	+
145	+	-	-
12	-	+	+
74	-	+	-
20	-	-	+
266	-	-	-
624			

UACS: Upper airway cough syndrome, GERD: Gastroesophageal reflux disease

Table S3. Triggers of cough in participants at follow-up

Triggers of cough	N
Throat itchiness	4806
Common cold	4470
Dust exposure	4047
Dry air	2010
Cold air	1991
Mold smell	1570
Running nose falls into back of the throat	1558
Pollen season	1530
Smoke or fragrance	1417
Spices	1165
Night, early morning	1137
Exercise	1112
Conversation or Laughing	676
Wet air	470
Exhaust, stress	434
Lie down	409
Contact with pets	267
Eating	170
Drinking alcohol	107

Table S4a. Comparative analysis of citric acid and new-onset prolonged cough/ prolonged cough at follow-up in participants with top 3 cough triggers without underlying diseases

	New-onset prolonged cough			Prolonged cough at follow-up		
	Yes<br>n = 296	No<br>n = 2757	p value	Yes<br>n = 427	No<br>n = 2936	p value
Citric acid	0.66 ± 0.12	0.68 ± 0.14	0.029	0.66 ± 0.13	0.68 ± 0.14	0.006

Table S4b. Multiple regression analysis of new-onset prolonged cough/prolonged cough at follow-up involving 4 metabolites in participants with top 3 cough triggers without underlying diseases

	New-onset prolonged cough		Prolonged cough at follow-up	
	OR (95% CI)	*P*-value	OR (95% CI)	*P*-value
Age, per 10-yr increase	0.94 (0.83–1.06)	0.32	0.89 (0.80–0.98)	0.02
Sex, female	1.38 (0.89–2.15)	0.15	1.40 (0.97–2.03)	0.07
BMI, per 2 kg/m^2	1.03 (0.94–1.13)	0.54	1.04 (0.97–1.13)	0.28
Ex smoking, %	0.88 (0.58–1.33)	0.54	0.88 (0.62–1.25)	0.48
%FEV_1, per 10%	0.94 (0.85–1.03)	0.18	0.94 (0.87–1.02)	0.13
Serum total IgE at baseline, IU/mL*	0.72 (0.55–0.94)	0.01	0.79 (0.64–0.99)	0.04
Blood eosinophil count, per 10/μL*	1.01 (0.99–1.02)	0.42	1.00 (0.99–1.01)	0.63
Citrus consumption	1.03 (0.71–1.49)	0.87	0.97 (0.71–1.32)	0.83
Blood collection time after meal, h	1.00 (0.97–1.04)	0.99	1.01 (0.98–1.04)	0.73
Citric acid, intensity *	0.09 (0.01–0.93)	0.04	0.09 (0.01–0.66)	0.02
Isocitric acid, intensity *	0.87 (0.19–4.10)	0.86	1.13 (0.31–4.12)	0.85
BHB, intensity *	1.04 (0.64–1.70)	0.88	0.83 (0.55–1.25)	0.36
HIBA, intensity *	1.45 (0.36–5.84)	0.60	1.31 (0.41–4.20)	0.65

*Log-transformed value was used for analysis.

Definition of abbreviations: BHB: 3-hydroxybutyric acid, BMI: body mass index, CI: confidence interval, FEV_1: forced expiratory volume in one second, HIBA: 3-hydroxyisobutyric acid, OR: odds ratio.

Figure S1.

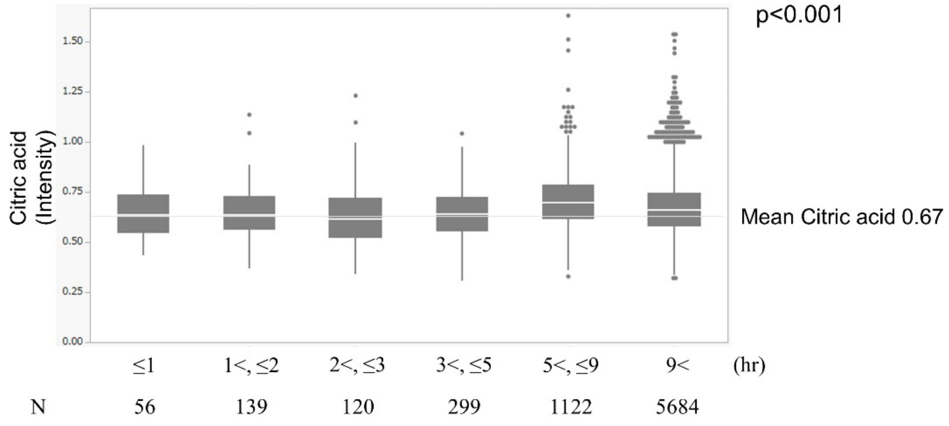

Figure S1**.** Plasma citric acid level according to postprandial period (missing data for 12 participants). One-way analysis of variance was conducted using log-transformed citric acid level. Horizontal line means mean of plasma citric acid. Median and quartiles are presented.

Figure S2a.

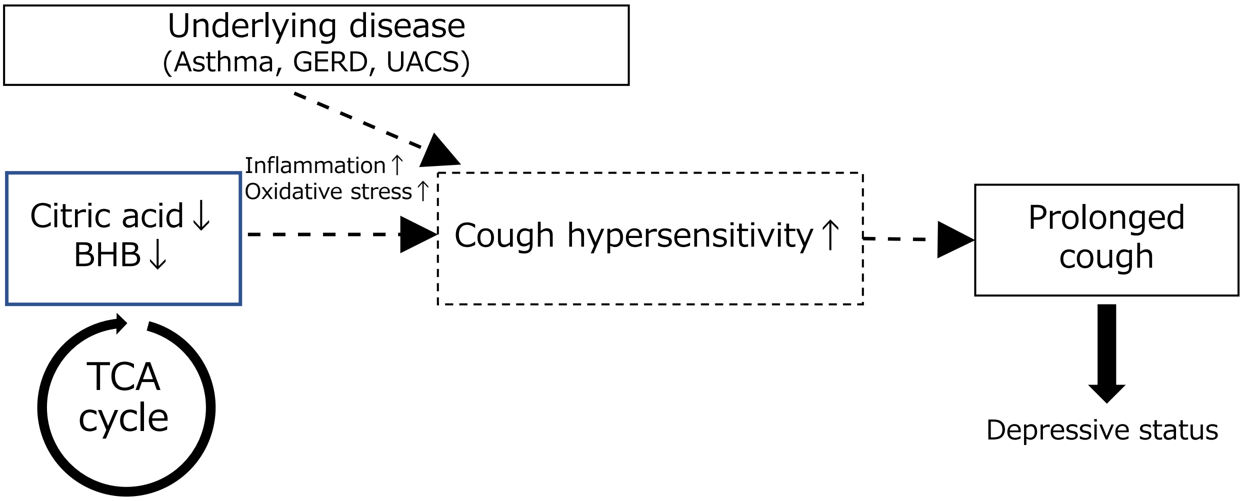

Figure S2a. Hypothesis about mechanisms of prolonged cough. Solid arrows indicate known causal relationships, and dotted arrows indicate assumptions based on this study. BHB: 3-hydroxybutyric acid, GERD: gastroesophageal reflux disease, TCA: tricarboxylic acid, UACS: upper airway cough syndrome.

Figure S2b.

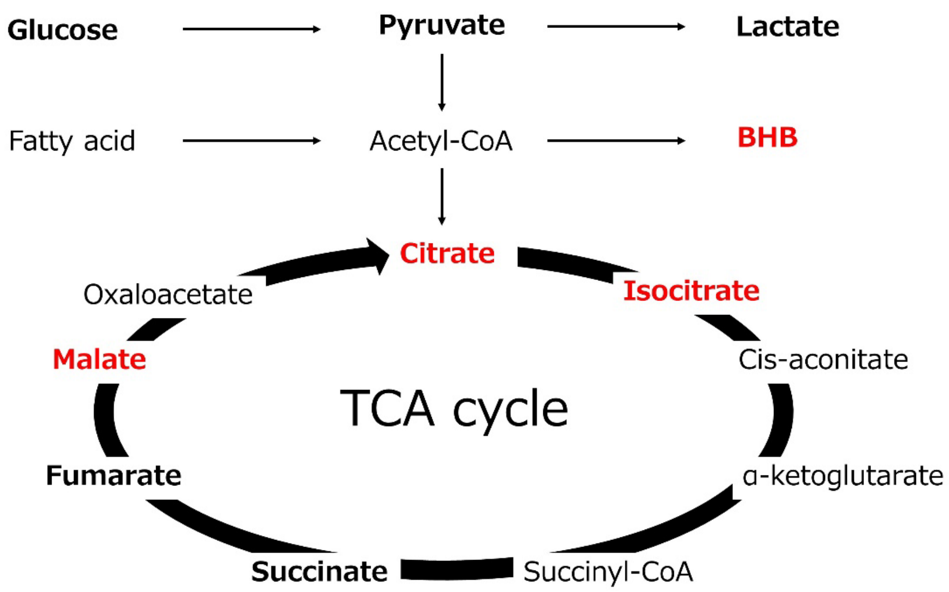

Figure S2b. Summary of TCA cycle-related metabolites measured in this study: measured metabolites are highlighted in bold. Metabolites potentially contributing to prolonged cough ($p < 0.1$ in Table 1b) are highlighted in red. BHB: 3-hydroxybutyric acid, TCA: tricarboxylic acid.
